# Decoding the genome and epigenome of avian *Escherichia coli* strains by R10.4.1 nanopore sequencing

**DOI:** 10.3389/fvets.2025.1541964

**Published:** 2025-03-19

**Authors:** Jingyao Wang, Xudong Liu, Yanwen Shao, Runsheng Li, Surya Paudel

**Affiliations:** Department of Infectious Diseases and Public Health, Jockey Club College of Veterinary Medicine and Life Sciences, City University of Hong Kong, Kowloon, Hong Kong SAR, China

**Keywords:** avian pathogenic *Escherichia coli*, chickens, nanopore, epigenetics, methylation

## Abstract

Avian pathogenic *Escherichia coli* (APEC) causes colibacillosis in poultry, which is a very important disease worldwide. Despite well-documented genomic traits and diversity of APEC, its epigenomic characteristics are less understood. This study utilized the high throughput and long-read capabilities of Oxford Nanopore Technology (ONT) to elucidate the genome structures and methylation modifications of three *E. coli* isolates of avian origin: one intestinal isolate from a healthy wild bird and two systemic isolates from clinically affected chickens. Three complete genomes, each comprising a single chromosome and multiple plasmids were assembled. Diverse virulence-associated genes, antimicrobial resistance genes, mobile genetic elements plasmids and integrons were characterized from the genomes. Despite a limited sample size, our whole genome sequencing (WGS) data highlighted significant genomic diversity among the *E. coli* strains and enriched repertoire of gene clusters related to APEC pathogenicity. From the epigenetic analysis, multiple methylation modifications, including three N5-methylcytosine (5mC), eight N6-methyladenine (6mA) and two N4-methylcytosine (4mC) modification motifs were identified within all three isolates. Furthermore, common GATC and CCWGG methylation motifs were predominantly distributed within regulatory regions, suggesting a role in epigenetic transcription regulation. This study opens the avenue for future research into pathogenesis, diagnostic and therapeutic strategies of APEC considering epigenetic analysis.

## 1 Introduction

*Escherichia coli*, a Gram-negative microorganism, is commonly found in the intestine of chickens as a normal inhabitant and has beneficial physiological impacts in growth and development (1, 2). However, some strains commonly called as avian pathogenic *E. coli* (APEC) can cause a wide variety of pathologies in poultry, such as omphalitis, salpingitis, peritonitis, airsacculitis, perihepatitis, and pericarditis, which are collectively called colibacillosis (3). Colibacillosis is a leading cause of increased mortality and morbidity as well as decreased egg quality and meat production, resulting in significant economic losses in poultry worldwide, which has negative influences on both productivity and animal welfare. The problem has been increasing in recent years due to changes in husbandry practices and strict legislation for antimicrobial use (4).

While many virulence factors have been identified in APEC isolates, no single gene or set of genes has been found to exclusively distinguish pathogenic from non-pathogenic avian *E. coli* isolates (5, 6). It is suggested that APEC isolates usually possess large, conjugative virulence plasmids belonging to the IncF incompatibility group (7, 8). APEC isolates harbor diverse combinations of virulence-associated genes (VAGs) involved in bacterial toxicity, adhesion, invasion, iron acquisition, antimicrobial resistance, survival, and metabolism under stress (9, 10). An additional complication is that clinical APEC isolates from colibacillosis cases exhibit remarkable genetic diversity, both across countries and within individual flocks or outbreaks (6, 11). The genetic heterogeneity and complexity are a major hindrance for the prevention of the disease by vaccination (12).

Despite extensive studies at the genomic level, the epigenomic characteristics of APEC remain underexplored. Bacteria have three common types of DNA methylation which are N6-methyladenine (6mA), N4-methylcytosine (4mC), and C5-methylcytosine (5mC). The methylation patterns are catalyzed by methyltransferase (MTase) enzymes that use S-adenosyl methionine to add a methyl group to the target DNA bases position (13, 14). MTase can couple with cognate restriction endonucleases to form RM (Restriction-Modification) systems in bacteria to safeguard host DNA sequences by cleavage and digestion of unmodified foreign DNAs but protecting their own methylated DNA. In addition to the RM system, bacteria also possess orphan methyltransferases that lack a corresponding restriction enzyme, such as the Dam family and the cell cycle-regulated CcrM methyltransferase (14, 15). There is emerging evidence that DNA methylation has roles in regulating signals of transcription, cell cycle control, new strand DNA repair and replication, phase variation and virulence expression (13, 16–18). Adenine methylation causes changes in DNA structure and influence DNA-protein interaction. In *E. coli*, methylation of GATC sites located in the consensus RNA polymerase binding region can repress transcription of *Tn10* transposase while activating transcription of *dnaA* by modifying interactions with regulatory proteins (19).

Deciphering the complete genome of pathogens is crucial for pathogenomics and virulome analysis to facilitate the development of effective vaccines and treatments. The challenges in plasmid genetic analysis and reconstruction arise when using short-read sequencing data due to rearrangements driven by recombination, architecture of repetitive elements including transposable elements (TEs), variations in gene copy numbers, and high sequence diversity (20). Furthermore, traditional sequencing technologies often cannot detect DNA methylation directly, limiting our understanding of epigenetic modifications in bacteria (13). However, Nanopore sequencing, a third-generation approach, offers long reads spanning repetitive regions, enabling complete plasmid structure determination, rapid turnaround time for timely analysis, real-time sequence analysis, and facilitating resistant gene detection. It is also a powerful technology for the detection of DNA modifications by measuring changes in ionic current as DNA molecules pass through nanopores (21). Compared with Pacific Biosciences (PacBio) (22), Oxford Nanopore Technologies base-calling systems can detect all three types of modification with equal efficiency. Therefore, nanopore sequencing is regarded as a valuable tool for comprehensive plasmid characterization and bacterial DNA methylation analysis (23–25). The R10.4.1 flow cell from ONT demonstrates exceptional modal accuracy exceeding 99%, providing superior performance in the detection of insertions, deletions, and homopolymer regions across a range of read lengths (26, 27).

In this study, we performed whole genome sequencing analysis of three *E. coli* isolates from varying clinical background, using ONT to decode their genomic and epigenomic profiles. In addition to detailed characterization of genetic traits of isolates such as genes, plasmids, virulence factors, antimicrobial resistance (AMR) genes, their methylome properties were investigated, which is not well understood in *E. coli* from poultry.

## 2 Materials and methods

### 2.1 *Escherichia coli* isolates and DNA extraction

The *E. coli* strains EC-O119H4 and EC-O117H42 were isolated from the liver and yolk sac of a 10-day-old chicken. The primary symptoms observed in the bird were anorexia, dry feet, and pasty vent. A mortality rate of approximately 30% was observed in the flock. The third isolate EC-O153H30 was obtained from the intestine with a cloacal swab from a healthy migratory wild bird (common greenshank). The pure culture of all the isolates were stored at −80°C in a glycerol solution until further processing. Afterwards, subculture was made on MacConkey agar plates and incubated at 37°C overnight. A single bacterial colony was picked and inoculated into Luria-Bertani (LB) broth, which was then incubated overnight at 37°C. The genomic DNA was extracted using MiniBEST Bacterial Genomic DNA Extraction Kit (TakaRa Bio Inc., Shiga, Japan) in accordance with the manufacturer’s instructions for sequencing.

### 2.2 Antimicrobial susceptibility test

The antimicrobial susceptibility test was done to assess the phenotypic sensitivity of *E. coli* isolates against several antibiotics using disk diffusion method. Briefly, the inoculums of the isolates were spread onto agar plates, antibiotics disks were placed, and incubated under aerobic conditions at 37°C for 24 h. The diffusion diameters were evaluated in accordance with the guidelines provided by the Clinical and Laboratory Standards Institute (CLSI) and categorized as resistant (R), susceptible (S), or intermediate (I).

### 2.3 Nanopore sequencing, assembly, and annotation

A library was constructed by ligation sequencing kit V14 SQK-LSK114 and whole-genome sequencing was performed on the ONT MinION platform with R10.4.1 flowcell FLO-MIN114 (Oxford Nanopore Technologies, UK, R10.4.1 FLO-MIN114). The raw signal in POD5 formed was basecalled using Dorado 0.8.0 with the SUP model named dna_r10.4.1_e8.2_400bps_sup@v5.0.0. Both simplex and duplex reads were basecalled. The simplex reads were used as the input for genome assembly. Flye 2.9.2 (28) was used for assembling the EC-O119H4 and EC-O153H30 strains, while Unicycle 0.5.1 (29) was employed for the EC-O117H42 strain. The raw assemblies were polished in three consecutive rounds with the simplex reads, followed by three additional rounds of polishing with duplex reads using Racon 1.5.0 (30). The closed genomes were annotated using the Prokaryotic Genome Annotation Pipeline (PGAP).

### 2.4 Bioinformatics analysis

Multilocus sequence typing (MLST) was performed using MLST 2.2.3^1^ and serotypes were identified by SerotypeFinder v.2.0.2 (31). Presence of AMR genes was identified using AMRFinderPlus v3.12.8 (32). The virulence genes were predicted using Diamond against the Virulence Factors Database (VFDB)^2^ (33). Only hits with at least 90% query and subject cover and identity 60%, along with *e*-values of 1e-5, were classified as virulence factors. Plasmid replicon types were identified using PlasmidFinder v2.1.6 (34). *Escherichia* genus strain phylotyping was performed with ClermonTyping 24.02 (35). Genovi was employed to generate visual maps of circular chromosome and plasmids (36). Genetic structure maps were generated using SnapGene^®^ software version 7.2.

### 2.5 Epigenetic analysis

In order to profile the epigenetic information in the *E. coli* strains, both Dorado 0.8.0 and Hammerhead 0.2.0 (37) were utilized. The modification proportion at each genomic site was determined using methylation models 4mC_5mC@V2 and 6mA@V2, which are compatible with the dna_r10.4.1_e8.2_400bps_sup@v5.0.0 in Dorado. The generated modBAM files then were profiled with modkit 0.4.1^3^ with functions “pileup” and “find_motifs.” In addition, the basecalled reads were processed with Hammerhead to locate potential modification sites, which were subsequently input into Meme 5.5.7 (38) for motif enrichment analysis. For metagene analysis, the modification distribution within gene body and upstream and downstream 200 bp were conducted by R script.

## 3 Results

### 3.1 Genome assembly, annotation, and serotypes of *Escherichia coli* strains

**Figure 1:**
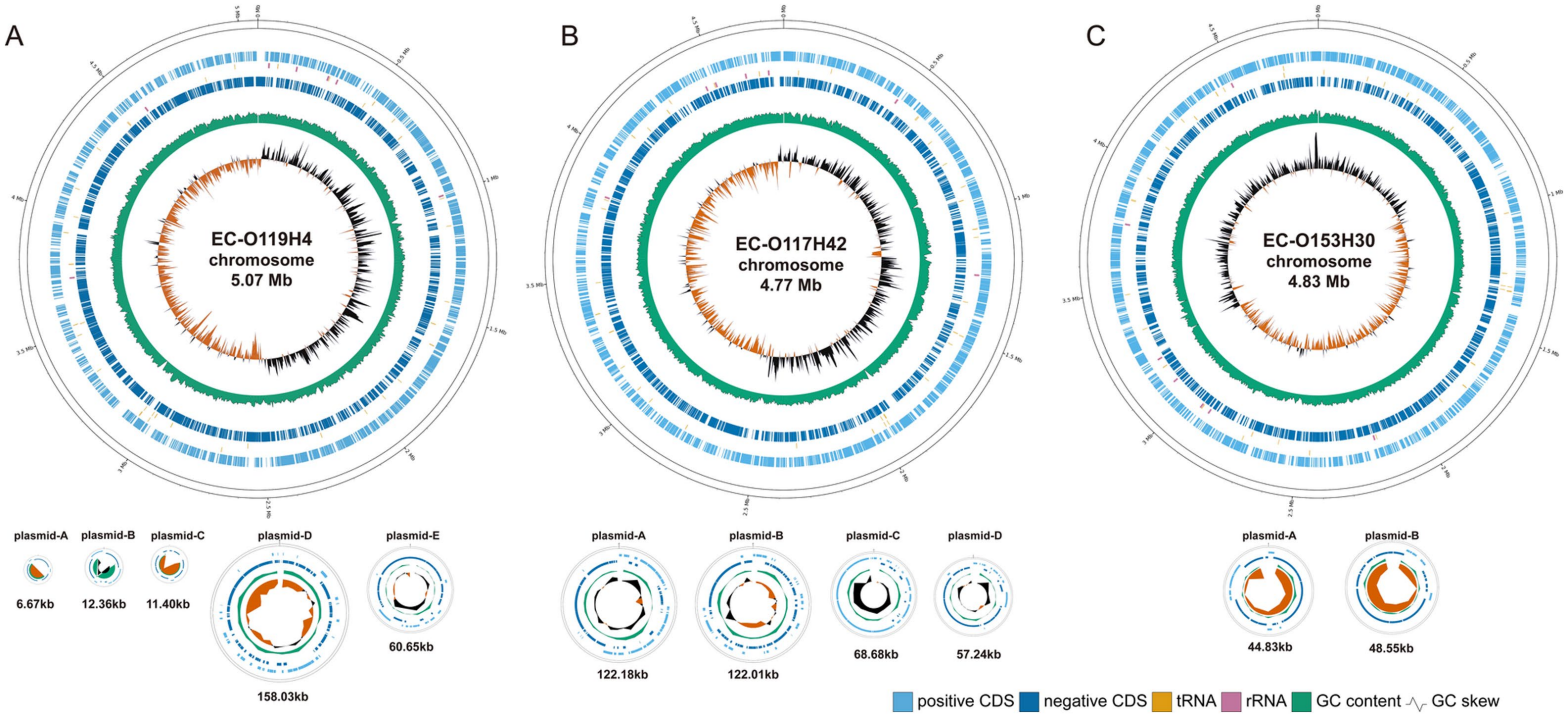
The circular genome map of EC-O119H4 **(A)**, EC-O117H42 **(B)**, and EC-O153H30 **(C)**. The circular genome of *E. coli* including chromosomes and plasmids visualized using GenoVi. From the innermost circles, Circle 1 shows the GC skew (G−C/G+C). The value is plotted as the deviation from the average GC skew of the entire sequence. Circle 2 shows the GC content, plotted using a sliding window. Circles 3 and 4 illustrate the coding sequences; 3 is negative, 4 is positive. The vertical bars near circles 3 and 4 represent corresponding RNA, with orange indicating tRNA and pink indicating rRNA. The chromosomes and plasmids are shown not to scale.

The ONT successfully generated three complete bacterial genome assemblies, with sequencing reads ranging from 295,955 to 562,655 and N50 values between 5,091 and 7,962. All three genomes were assembled into a single circular chromosome measuring 4.77 to 5.07 mb, along with varying numbers of plasmids ranging from 6.67 to 158 kb in length (Table 1; Figure 1).

**Table 1:** Overview of genome assembly, genomic features of *Escherichia coli* strains EC-O119H4, EC-O117H42, EC-O153H30.

Genomic traits	EC-O119H4			EC-O117H42		EC-O153H30	
	Chromosome	Plasmid	Plasmid-IncF	Chromosome	Plasmid	Chromosome	Plasmid
GenBank accession no.	CP162393	CP162394, CP162395, CP162396, CP162398	CP162397	CP172331	CP172332, CP172333, CP172334, CP172335	CP172336	CP172337, CP172338
Size (bp)	5,067,238	6,670, 12,360, 11,394, 60,653	158,031	4,772,837	122,178, 122,007, 68,683, 57,243	4,832,895	44,831, 48,547
GC content (%)	50.78	56, 50.33, 49.67, 42.31	50.25	50.71	48.68, 51.56, 52.29, 42.25	50.66	48.56, 44.9
No. of coding sequence	4,841	6, 11, 11, 77	180	4,568	135, 132, 80, 72	4,480	65, 69
No. of RNAs	tRNA (90), rRNA (22)	–	–	tRNA (87), rRNA (22)	–	tRNA (85), rRNA (22)	– tRNA (1)
Replicon	–	Col156, − Col156, IncI2(Delta)_1	IncFIA, IncFIB, IncFII	–	IncI1, IncY, IncFII, IncI2	–	–
Antimicrobial resistance (AMR) genes	*cyaA* [S352T], *gyrA* [S83L], *glpT* [E448K], *parC* [S80I]	–	*aac(3)-IId*, *aadA2*, *blaTEM-1*, *lnu(F)*	*blaCTX-M-55*, *tet(A)*, *aph(6)-Id*, *aph(3″)-Ib*, *sul2*, *fosA3*, *parC* [S80I], *gyrA* [S83L]	*dfrA14*, *floR*, *aph(3′)-Ia*, *qnrS1*, *arr-2*, *blaTEM-1*, *aph(6)-Id*, *aph(3″)-Ib*, *sul2*, *aac(3)-IId*, *lnu(F)*, *aadA2*, *tet(A)*	*blaCTX-M-15*, *qnrS1*, *tet(B)*, *glpT* [E448K], *cyaA* [S352T], *uhpT* [E350Q]	–

Among two strains obtained from diseased chicken, EC-O119H4 from the liver, was classified as ST117 and belonged to the phylogenetic group F and the serotype O119:H4. The other strain, EC-O117H42, isolated from the yolk sac, was identified as the O117:H42 serotype, classified as ST2207, and assigned to the phylogenetic group A.

The EC-O119H4 genome consisted of 5,126 protein-coding sequences (CDS), 90 tRNA genes and 22 rRNA genes. It contained five plasmids, with the largest pEC-O119H4-D-IncF identified as a hybrid IncFIB/IncFIA/IncFII replicon was characterized by the presence of *repB*, *repA*, and *RNAI-FII* sequences. This plasmid includes 180 coding sequences and has a GC content of 50.25% (Table 1). Additionally, two plasmids with the Col156 replicon and one with the IncI2 replicon were identified.

The EC-O117H42 genome encoded 4,987 protein-coding sequences (CDS), 87 tRNA genes, and 22 rRNA genes. Along with an IncFII plasmid, three additional plasmids were found, carrying replicons IncI1, IncY, and IncI2 (Table 1).

The intestinal *E. coli* strain EC-O153H30, was classified into serotype O153:H30, and belonged to the phylogenetic group F and sequence type ST12280. The chromosome contains 4,480 protein-coding sequences (CDS), 85 tRNA genes, and 22 rRNA genes (Table 1). Notably, two plasmids in CE-O153H30 did not produce any identifiable amplicons.

### 3.2 Virulence-associated genes profiles

In the chromosome of EC-O119H4, P fimbriae genes *papBCDEFGIJK*, type I fimbriae genes *fimABCDEFGHI*, curli fiber genes *cgsDEFG* and *csgCDEFG*, common pilus genes *yagVWXYZ/ecpEDCBA*, *ykgK/ecpR* and chemotaxis genes *cheBDRWYZ* for adherence were found. Likewise, heme uptake genes, *chuASTUVWXY*, yersiniabactin siderophore genes *fyuA*, *irp2*, *ybtAETQU*, and enterobactin siderophore genes *entABCDEFS, fes*, *fepBCDEG* were also identified (Supplementary Table S1). Additionally, the genome harbored genes associated with toxins (*pic*), invasion (*ibeBC*, *ompA*), colanic acid production (*ugd*), oxidative pentose phosphate metabolic pathway gene (*gnd*) and type VI secretion system.

The EC-O117H42 chromosome also contained well-known virulence factors, including type I fimbriae (*fim* family), curli fibers (*csg* genes and *cgs* genes), and the common pilus (*yag*/*ecp* genes cluster) for colonization (Supplementary Table S1). Additionally, *ibeBC* and *ompA* for invasion, and enterobactin system for iron uptake, *ugd* for the synthesis of UDP-glucuronic acid and *gnd* for 6-phosphogluconate dehydrogenase were found. Compared to EC-O119H4 isolate, this strain lacks P fimbriae, yersiniabactin system and heme transport system.

The intestinal *E. coli* isolate EC-O153H30 features a chromosome that includes common type 1 fimbriae (*fim* cluster) and *ompA*, facilitating adhesion. Furthermore, it contains other prevalent virulence factors distinct from the two clinical strains, including a type III secretion system and the K1 capsule (*kpsCDEGMSTU*), which contributes to anti-phagocytic activity. Concerning iron utilization, it encodes the biosynthesis of enterobactin (*ent* genes, *fes*, *fep* genes) and heme absorption (*chu* cluster) (Supplementary Table S1).

In the IncF plasmid of the EC-O119H4, many genes linked to APEC virulence, including salmochelin siderophore system *iroBCDE*, increased serum survival gene *iss,* iron transport genes *sitABCD*, arginine deiminase operon *arcAC* and aerobactin uptake genes *iucABCD*/*iutA* were found (Supplementary Figure S1). In contrast, while EC-O117H42 also possesses an IncF plasmid, it does not contain any virulence genes. The EC-O153H30 isolate lacks the large IncF plasmid, which is a low-copy number conjugative plasmid ranging in size from 45 to 200 kb, typically containing aerobactin iron acquisition systems and factors for serum survival.

### 3.3 Genotypic and phenotypic antimicrobial resistance

In EC-O119H4 strain, the AMR genes, including *lnu(F)*, *aadA2*, *aac(3)-IId*, *blaTEM-1* are located on the IncF plasmid between positions 89 and 98 kb, flanked by an IS26 transposase and an IS1 family transposase (Supplementary Figure S1). Antimicrobial susceptibility test showed that the EC-O119H4 was resistant to amoxicillin, enrofloxacin, tilmicosin, whereas susceptible to florfenicol, kanamycin, ciprofloxacin, chloramphenicol. The resistance phenotype for amoxicillin correlates well with the resistance genotype *blaTEM-1* (beta-lactam resistance). Likewise, the SNPs in *gyrA* and *parC* that confer resistance to fluoroquinolones correlates with phenotypic resistance to enrofloxacin. Nonetheless, *lnu(F)*, *aadA2*, *aac(3)-IId* as well as *cyaA*_S352T and *glpT*_E448K, do not show a direct correlation with resistance phenotypes for tilmicosin. This may be because many antimicrobial resistance (AMR) proteins can reduce antibiotic susceptibility to some extent but not enough to surpass clinical breakpoints. Additionally, an isolate may acquire or lose resistance to an antibiotic through mutational processes, such as the deletion of a porin required for the antibiotic to penetrate the cell. The EC-O119H4 isolate harbors a class 1 integron that carried genes *lnu(F)*, *aadA2*, flanked by IS26 transposase (Supplementary Figure S1). The BLAST search reveals this class 1 integron had high sequence identity (>99.9%) and 100% coverage with plasmid p13C1065T-2 integron from Hong Kong (GenBank accession number: CP019261.1), and a *Salmonella typhimurium* strain plasmid from Guangdong (GenBank accession number: AP027789.1).

Similarly, EC-O117H42 was also found to carry multiple resistance genes, including ESBL-producing *blaCTX-M-55*, aminoglycoside O-phosphotransferase *aph(6)-Id* and *aph(3″)-Ib*, amino acid substitution in *parC* and *gyrA* associated with quinolone resistance, tetracycline resistance gene *tet(A)*, sulfonamide resistance *sul2*, fosfomycin resistance *fosA3*. Plasmid pAPEC-O117H42-B contains antimicrobial resistance genes, including *dfrA14*, *floR*, *aph(3′)-Ia*, *qnrS1*, *arr-2*, *blaTEM-1*, *aph(6)-Id*, *aph(3″)-Ib*, *sul2*, *aac(3)-IId*, *lnu(F)*, *aadA2*, and *tet(A)*, which confer resistance to trimethoprim, phenicol, aminoglycosides, quinolones, rifampin, beta-lactam antibiotics, sulfonamides, and tetracycline (Table 1). Susceptibility testing results indicated that EC-O117H42 was resistant to all tested antibiotics, including amoxicillin, enrofloxacin, florfenicol, kanamycin, tiamulin, ciprofloxacin, and chloramphenicol, demonstrating a direct correlation with the resistance genotypes. It contains a class 1 integron that carries the genes *lnu(F)* and *aadA2*, demonstrating 99.96% sequence identity and 100% coverage with the class 1 integron of pEC-O119H4-D-IncF.

The intestinal *E. coli* strain EC-O153H30 harbors three AMR genes located on its chromosome, including *blaCTX-M-15*, *qnrS1*, and *tet(B)* (Table 1). These genes are responsible for resistance to beta-lactams, quinolones, and tetracyclines, respectively. Additionally, the EC-O153H30 strain has three mutation sites in *glpT* [448: E-K], *cyaA* [352: S-T], and *uhpT* [350: E-Q]. The antimicrobial susceptibility testing revealed that EC-O153H30 exhibited resistance to tetracyclines and cefpodoxime, which aligns with its genotype.

### 3.4 Description of methylome profiles in *Escherichia coli* strains

**Figure 2:**
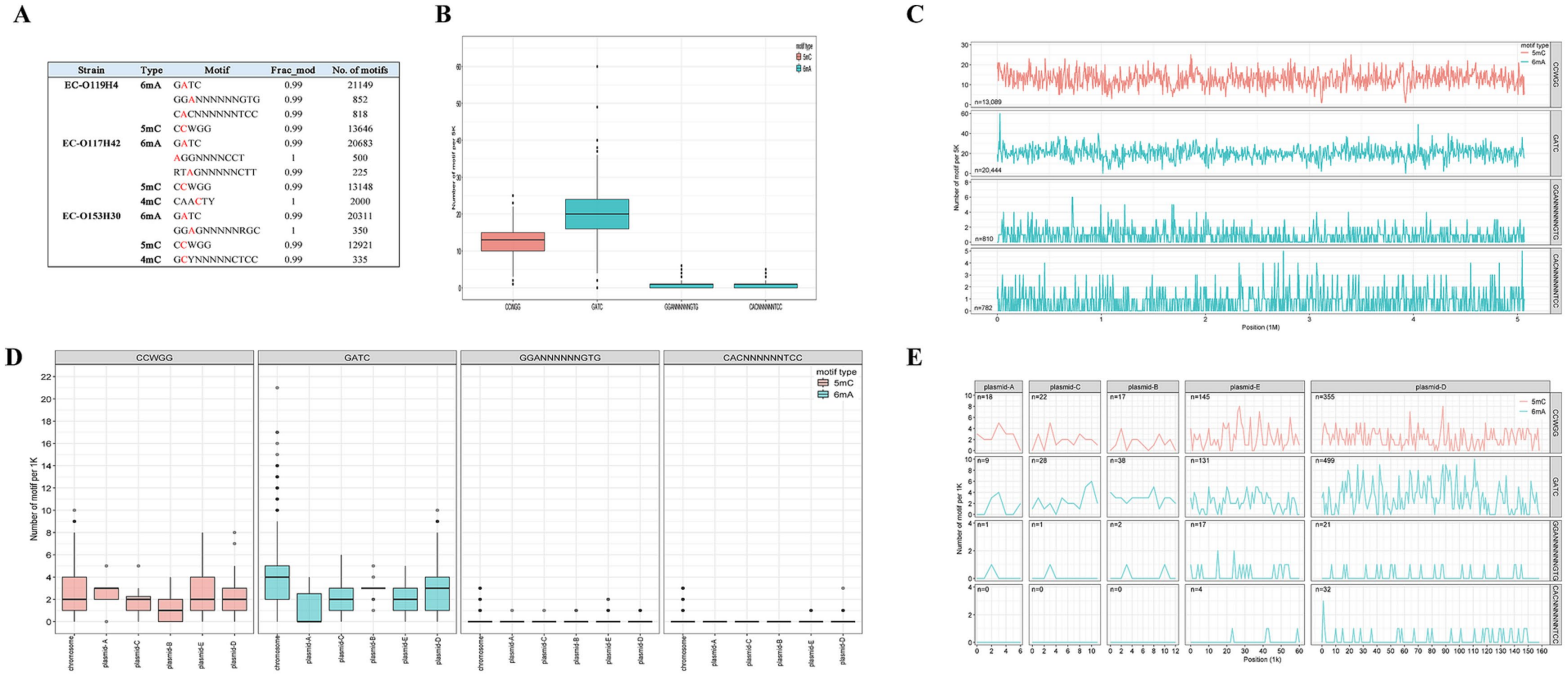
DNA modification within three *E. coli* strains determined by ONT sequencing **(A)** and DNA methylome across *E. coli* EC-O119H4 chromosome and plasmids **(B–E)**. Box plots **(B)** and frequency distribution **(C)** show the relative methylation levels of 5mC and 6mA motifs per 5 k bin size in the chromosome. Box plots **(D)** and frequency distribution **(E)** illustrate the relative methylation levels of 5mC and 6mA motifs for each 1 k bin size in the plasmids.

The methylated positions across the genome were detected using ONT sequencing. The EC-O119H4 strain has one conserved 5mC modification motifs CCWGG, and three conserved 6mA modification motifs GATC, GGANNNNNNGTG, and CACNNNNNNTCC (Figure 2A). Three modification types were identified in the genome of EC-O117H42 isolate, comprising 5mC modifications within CCWGG motif, 6mA modification residues within GATC, AGGNNNNCCT, RTAGNNNNNCTT motifs, and 4mC-modified cytosines. In EC-O153H30, 6mA is present in GATC, GGAGNNNNNRGC motifs, with 5mC methylated in motif CCWGG and 4mC methylated in motif GCYNNNNNCTCC. Notably, 99% of available sites are modified, which aligns with RM (Restriction-Modification) protection against cleavage by cognate restriction endonucleases.

To assess the distribution of 6mA, 5mC, and 4mC sites across the genome, we quantified the modification motifs in 5,000 bp regions in the chromosome and in 1,000 bp regions in the plasmids. Among the 13 methylation motifs identified in the three strains, GATC and CCWGG are shared by all and exhibit the most extensive distributions in each strain (Figure 2A). Each strain has over 20,000 GATC motifs and more than 10,000 CCWGG motifs, with GATC motifs being more prevalent than CCWGG motifs on the chromosome (Figures 2A–C; Supplementary Figures S2, S3). Compared to EC-O119H4, the strains EC-O117H42 and EC-O153H30 exhibit 4mC modification motifs at lower levels (Figure 2A). EC-O119H4 exhibits higher methylation levels for all identified motifs compared to EC-O117H42 and EC-O153H30 (Figure 2A). Other identified methylated motifs show variability among different strains and appear to be uniquely possessed by specific *E. coli* strains (Figure 2; Supplementary Figures S2, S3).

**Figure 3:**
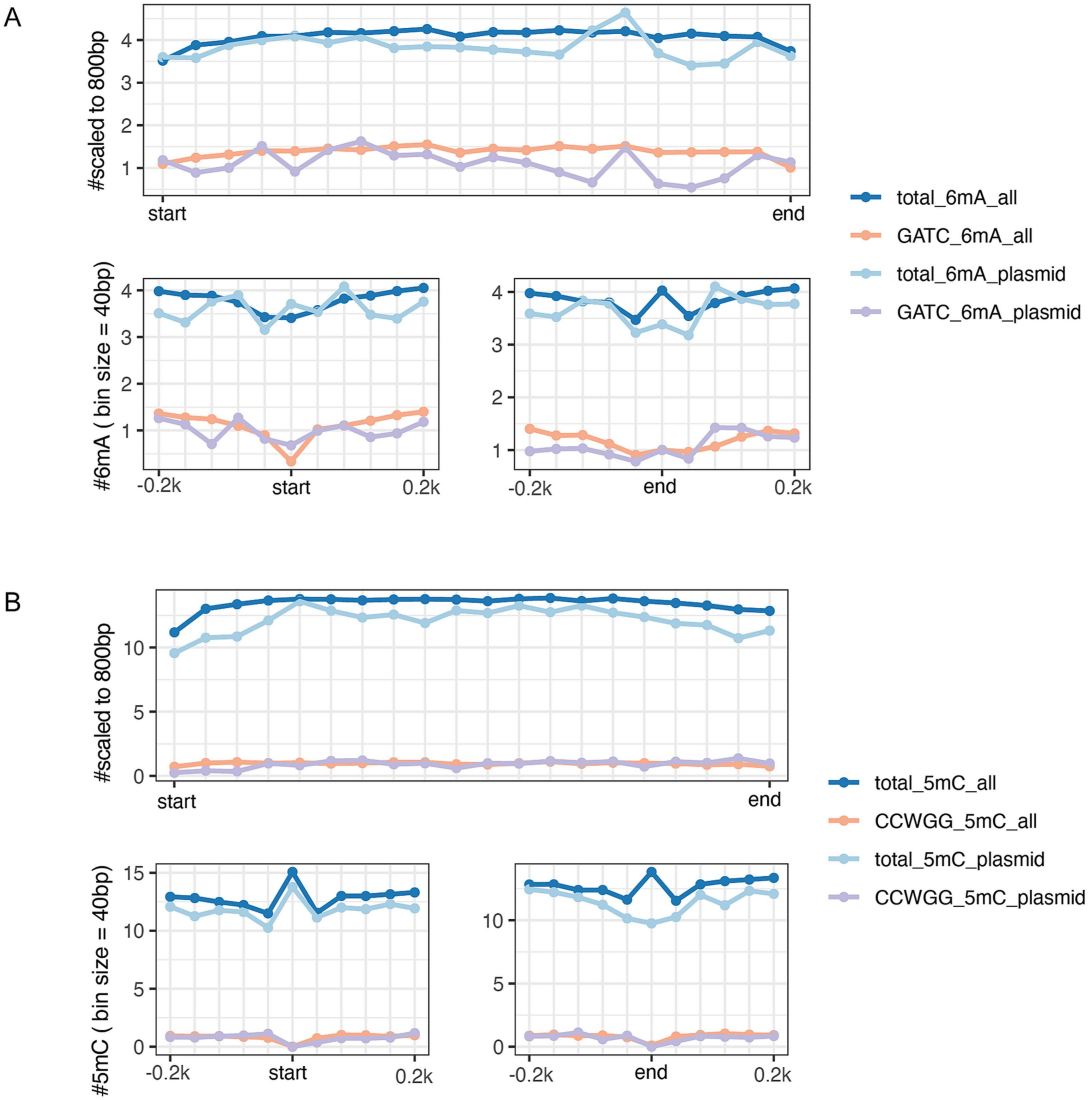
DNA methylation patterns of genes bodies (top panel) and at the gene start and end sites within 200 bp (bottom panels) in GATC motifs and total 6mA **(A)**, CCWGG motifs and total 5mC **(B)**. Deep Blue and orange lines refer to the genes that are sourced from entire genome both the chromosome and plasmids of EC-O119H4 isolate. The light blue and purple colors represent all genes solely from pEC-O119H4-D-IncF plasmid.

**Figure 4:**
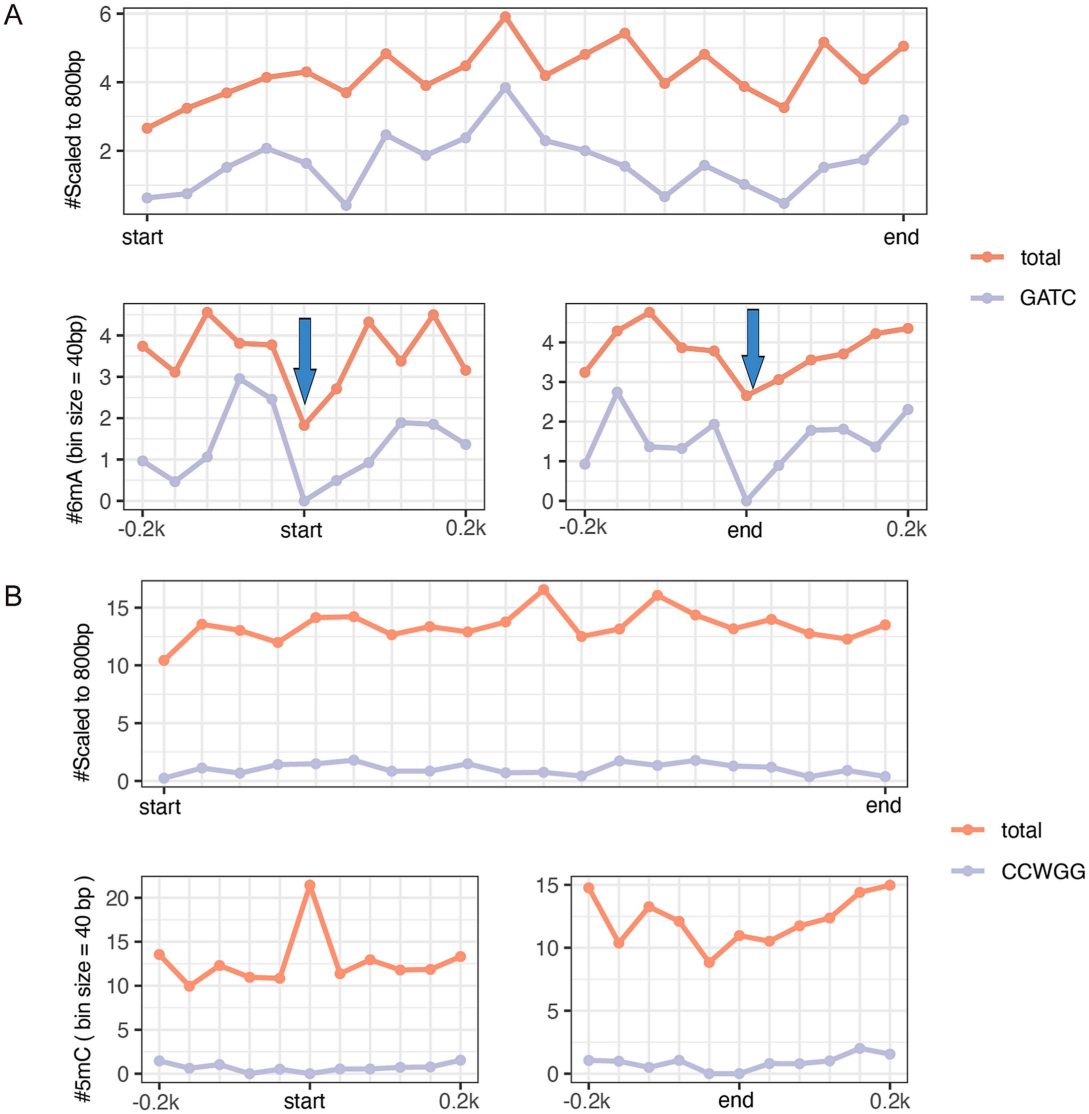
The metagene analysis of 18 virulence-associated genes and antimicrobial resistance genes (shown in Supplementary Table S2) in the pEC-O119H4-D-IncF plasmid per 40-bp bin size. The average GATC motifs and total 6mA across all gene bodies (top panel) and at the gene start and end sites within 200 bp (bottom panels) **(A)**. Arrows indicate a clear reduction in m6A frequency near the start and end coordinates. The average CCWGG motifs and total 5mC across all gene bodies (top panel) and at the gene start and end sites within 200 bp (bottom panels) **(B)**.

Among the three *E. coli* isolates, only EC-O119H4 was found to contain the IncF plasmid, which is potentially promising candidate associated with higher pathogenicity of *E. coli* isolates in poultry (8, 10). Therefore, the EC-O119H4 strain was further analyzed for its methylation patterns. A metagene analysis of 6mA and 5mC density for its entire genome (including both chromosome and plasmids), the IncF plasmid, and the selected important 18 VAGs, and AMR genes was performed (Supplementary Table S2) on the IncF plasmid across three regions: the 200 bp upstream region (USR), the coding sequence (CDS), and the 200 bp downstream region (DSR). The methylated 5mC and 6mA displayed a modest density within gene bodies, regardless of whether the genes were located on plasmids or the genome (Figures 3, 4). A moderate decrease in GATC frequency was observed near the transcriptional start sites (TSSs) of genomic genes, whereas this trend was not evident in plasmid genes (Figure 3A). The analysis of 18 important genes on plasmid showed that both 6mA and GATC motifs were significantly decreased at the transcriptional start sites (TSSs) and termination sites (TESs) (Figure 4A). An increase in 5mC frequency was observed at the transcriptional start sites (TSS) across the genome, plasmid, and the 18 key genes within the plasmid, while no signs of the CCWGG motif are detected (Figures 3B, 4B). It was also found that the 3′ downstream regions of the total genomic genes exhibited hypermethylation for both 5mC and 6mA (Figure 3), while hypomethylation was observed for 6mA and GATC in 18 key genes within the plasmid (Figure 4A).

## 4 Discussion

*Escherichia coli* isolates from poultry exhibit a significant genomic plasticity and complexity. In this study, we utilized Nanopore Sequencing technology to analyze the comprehensive genomic and epigenomic traits in *E. coli* from systemic organs of a diseased chicken. Additionally, an intestinal isolate from a healthy wild bird was included to compare poultry *E. coli* isolates with those from a significantly different environment.

The diverse set of virulence genes suggests that the *E. coli* strains included in this study are well-equipped to mediate adherence, invasion and survival inside host cells, sequestering iron ions for colonization and growth, resistance to serum bactericidal activity, biofilm formation, contributing to its pathogenicity in avian hosts. The pathogenicity of APEC strains appears to be closely linked to plasmid-encoded genes such as *iroN*, *ompT*, *hlyF*, *iutA*, and *iss* (10), as well as the number and combination patterns of VAGs (39, 40). One of the common patterns in the virulence genotype of APEC strains reported so far is the presence of large IncF or ColV plasmids that harbor multiple genes responsible for three key functions: serum survival/complement resistance (*iss* and episomal *ompT*), iron acquisition (*iucD*, *iutA*, *iroN*, and *sitA*), and secretion (episomal/chromosomal *ompT*, *hlyF*, *tsh*, and *cva*/*cvi*). Chromosomal genes that show differential distribution between APEC and nonpathogenic *E. coli* such as *papC*, *papA*, *papG*, *fimC*, *tia*, and *ibeA* are primarily associated with the adhesion and invasion of *E. coli* into host cells (41). However, no single set of virulence genes is exclusively responsible for causing colibacillosis (6, 42, 43). Variations in 13 genes, including *yciC*, *group_2364* (*ISKpn28*), *iroE*, *iroN*, *iroB*, *fes*, *btuD*, *iss*, *group_180* (*ISKpn28*), *ompD*, *ompT*, *group_6989* (hypothetical protein), and *hlyF*, as well as SNPs in three different genes identified through genome-wide association study GWAS, appear to be associated with pathogenicity in APEC isolates (6).

The conserved motifs detected in different *E. coli* lineages can vary. The reference *E. coli* has been found to have 6mA at 39,872 GATC sites, 337 AAGANNNNNCTC sites, and 337 GAGNNNNNTCTT sites (44). A total of 49,311 6-methyladenine (6mA) residues including GATC, ACCACC, CCACN8TGA(T/C), TCAN8GTGG, CTGCAG, and 1,407 putative 5-methylcytosine (5mC) residues including CCWGG were identified in the whole genome of hemolytic uremic syndrome (HUS)-associated *E. coli* O104:H4 (45). Among the three methylation types in *E. coli*, 4mC occurs less frequently than 6mA and its function largely remains unclear (46, 47). Methylation by 5mC has been linked to Tn3 transposition and the expression of ribosomal proteins during the stationary phase, emerging as roles in virulence and host adaptation (48–50). Methylation of 6mA can induce transcriptional changes in response to various growth stages and environmental stimuli, depending on clock-like controls and switch-like controls, where transcription is regulated by the binding of transcription factors or RNA polymerase according to DNA methylation pattern (19, 51). In *E. coli*, genes associated with virulence traits can be regulated by DNA adenine methylation, as shown earlier with *pap* pilus family and phase-variable autotransporter protein gene *agn43* (19). Our data suggest that 6mA methylation may be associated with gene expression and their relationship are complex, depending on sequence context. Similarly, Casselli et al. also observed apparent reduction in m6A frequency near the start and end coordinates. Although there was no direct correlation detected between m6A distribution and transcriptome altering in *Borrelia burgdorferi*, Casselli et al. suggested only a subset of m6A modifications may have meaningful implications for gene expression changes (52), which is consistent with our perspective. It has been reported that the distribution of N6-methyladenine impacts gene expression and functionality in *Aeromonas veronii* and *Helicobacter pylori* (53). Notably, unmethylated motifs are particularly enriched in the promoters of functionally related genes especially transcriptional regulators, compared to methylated motifs, highlighting their potential involvement in regulatory processes (15). Interestingly, 6mA and 5-methylcytosine (5mC) exhibit opposite trends near the transcriptional start sites of key plasmid genes, suggesting their independent roles in regulating gene expression. This observation aligns with similar phenomena reported in fungi (54). However, the 5mC mentioned here does not correspond to the well-documented CCWGG motif but involves other 5mC sequences that remain to be fully explored. In the 3′ downstream regions near termination sites, 6mA displays hypermethylation across the genome, whereas key plasmid genes show hypomethylation of 6mA. This indicates that downstream regions of genes with 6mA may also contribute to the regulation of plasmid gene expression. The methylation of 6mA appears to be context-dependent with respect to sequence specificity. Although this study highlights the potential roles of 6mA and 5mC in epigenetic regulation of transcription, further experiments, such as deletion of the restriction-modification (RM) system and RNA sequencing (RNA-seq), are required to validate the impact of methylation on virulence gene expression in future investigations.

Several reports suggest that DNA methylation plays a critical role of regulating bacterial gene expression and virulence, enabling adaptation to the harsh environmental changes and modulating the interaction with the host (14, 55). Specifically, DNA methylation regulates various genes, structures, and processes associated with pathogenesis, such as virulence, host colonization, biofilm formation, adhesins, pili, iron transport proteins (56, 57). The essential role of DNA adenine methylase (Dam) in gene expression and cellular adhesion in uropathogenic *E. coli* (UPEC) was confirmed through comparative analysis of Dam mutants (58). The posttranslational methylation of flagellin of *Salmonella Typhimurium* facilitates adhesion to host cell surface and host infection (59). Fur and Dam compete at the −10 transcriptional element to finely tune the expression of type VI secretion system (T6SS) of enteroaggregative *E. coli* (60). Recent studies highlight that bacteria can alter the epigenotype and gene expression profiles of host cells, significantly contributing to pathogenesis (61–63). Bacterial-induced epigenetic disruptions can influence host cell functions, either enhancing host defense mechanisms or facilitating pathogen persistence. Moreover, recent research has identified a link between DNA methylation and multiple antimicrobial resistance (64). The relative positioning of DNA methylation may also provide insights into its functional role. A decrease in GATC frequency near TSSs in the genomic genes of *E. coli* in this study indicated that reduced GATC motifs primarily originate from the chromosome. However, when analyzing 18 important genes on plasmid, we found that both 6mA and GATC motifs were significantly decreased at the transcriptional start sites (TSSs) and termination sites (TESs) which indicates that only a crucial and small portion of genes on the IncF plasmid possess hypomethylated promoter regions. This key subset of genes on plasmid was associated with virulence and antimicrobial resistance (AMR). The decrease at the transcriptional start sites of key genes may implicate the epigenetic regulation of transcription associated with GATC. The hypermethylation of the 3′ downstream regions of the total genomic genes for both 5mC and 6mA, and in contrast the hypomethylation for 6mA and GATC in 18 key genes within the plasmid suggests that the methylation of upstream and downstream gene regions for 5mC and 6mA may collaborate to regulate gene expression, and methylation of 6mA is context-dependent on the sequence. In the actual study, no functional analysis was performed corelating methylation with the pathogenic potential of *E. coli* isolates, however the observed genetic and epigenetic factors may open the avenues for future studies to investigate the potential contribution of DNA modifications on bacterial pathogenesis and the chromatin-based regulation of defense genes as well as in proposing polyvalent vaccines, drugs, and diagnostic tools by targeting key invasive strategies, such as immune evasion, iron acquisition, adherence, and toxin production, to prevent bacterial infections.

## Data Availability

The original contributions presented in the study are publicly available. These data can be found in the NCBI repository: Bioproject: https://www.ncbi.nlm.nih.gov/bioproject/?term=PRJNA1081610 Sequence: https://www.ncbi.nlm.nih.gov/nuccore/CP172336.

## Footnotes

^1^ https://github.com/tseemann/mlst

^2^ http://www.mgc.ac.cn/VFs/

^3^ https://github.com/nanoporetech/modkit
