# Supplementary material for: Decoding the genome and epigenome of avian Escherichia coli strains by R10.4.1 nanopore sequencing
Source: Front Vet Sci. 2025 Mar 19;12:1541964. doi: 10.3389/fvets.2025.1541964 (PMC11963381; doi:10.3389/fvets.2025.1541964)
Supplement: Supplementary file 1 [file Data_Sheet_1.DOCX]

Supplementary Material

**Table S1**

Detailed list of virulence genes and their corresponding VF classes identified in *E. coli* isolates

**Figure S1**

**Figure S1:** The structure of IncF plasmid (pEC-O119H4-D-IncF) (GenBank accession number: CP162397). The sizes and orientations of the ORFs in the genome are indicated by arrows. Antimicrobial resistance genes are marked in red, transposases in green, virulence associated genes in pink, conjugal transfer genes in blue, and other genes are highlighted in grey.

**Table S2**

List of 18 genes linked to virulence and antimicrobial resistance of pEC-O119H4-D-IncF plasmid for metagene methylation analysis

**Figure S2**

**Figure S2:** The DNA methylome landscape of *E. coli* EC-O117H42. Box plots (A) and frequency distribution (B) of 5mC, 6mA, 4mC motifs per 5k bin size in the chromosome of EC-O117H42. Box plots (C) and frequency distribution (D) of 5mC, 6mA and 4mC motifs for each 1k bin size on the plasmids of EC-O117H42.

**Figure S3**

**Figure S3:** The DNA methylome landscape of *E. coli* EC-O153H30. Box plots (A) and frequency distribution (B) of 5mC, 6mA, 4mC motifs per 5k bin size in the chromosome of EC -O153H30. Box plots (C) and frequency distribution (D) of 5mC, 6mA and 4mC motifs for each 1k bin size on the plasmids of EC -O153H30.
