# Supplementary figures and images for: Decoding the genome and epigenome of avian Escherichia coli strains by R10.4.1 nanopore sequencing
Source: Front Vet Sci. 2025 Mar 19;12:1541964. doi: 10.3389/fvets.2025.1541964 (PMC11963381; doi:10.3389/fvets.2025.1541964)

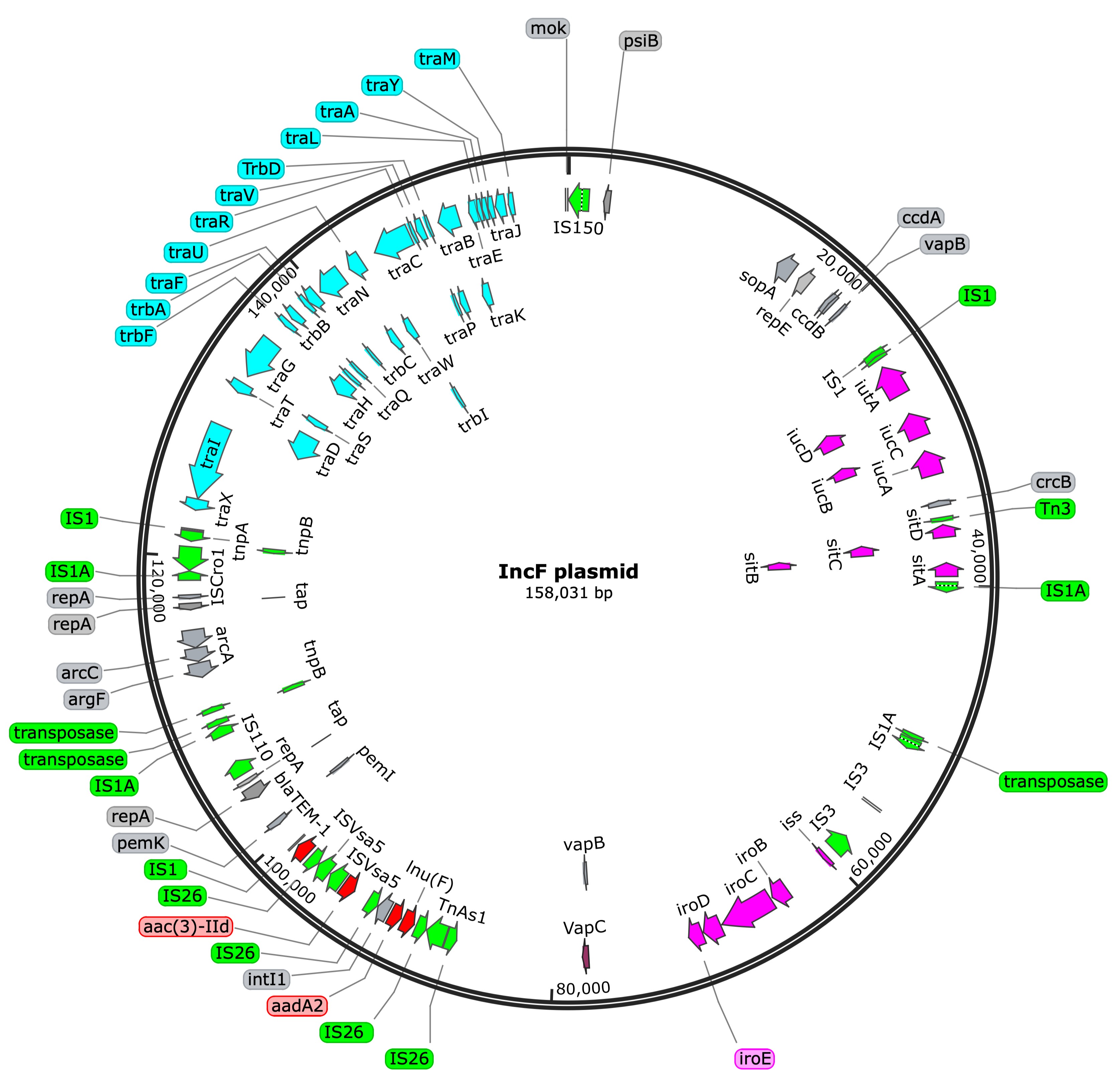

Supplement: Supplementary file 2 [file Image_1.JPEG]

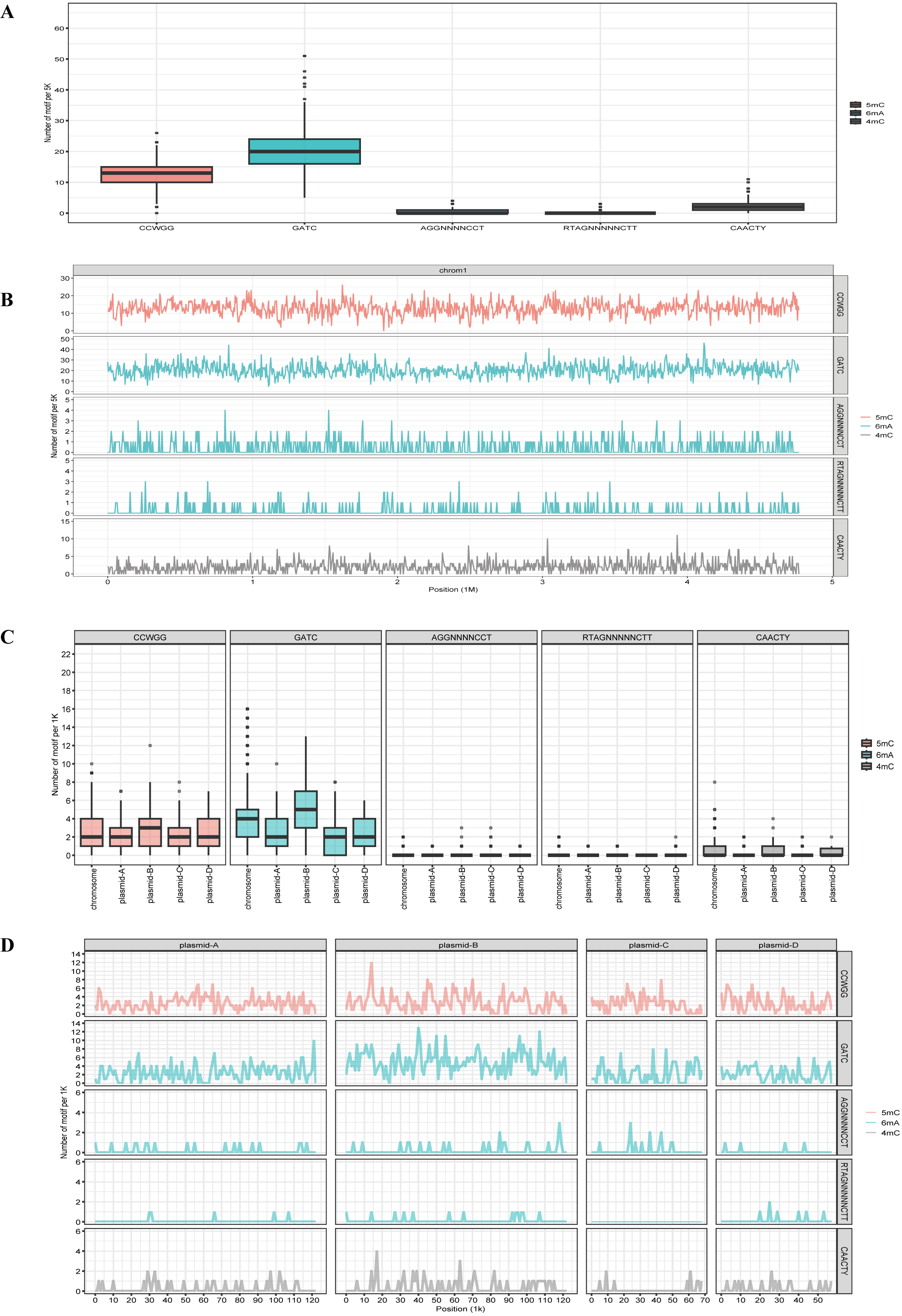

Supplement: Supplementary file 3 [file Image_2.TIF]

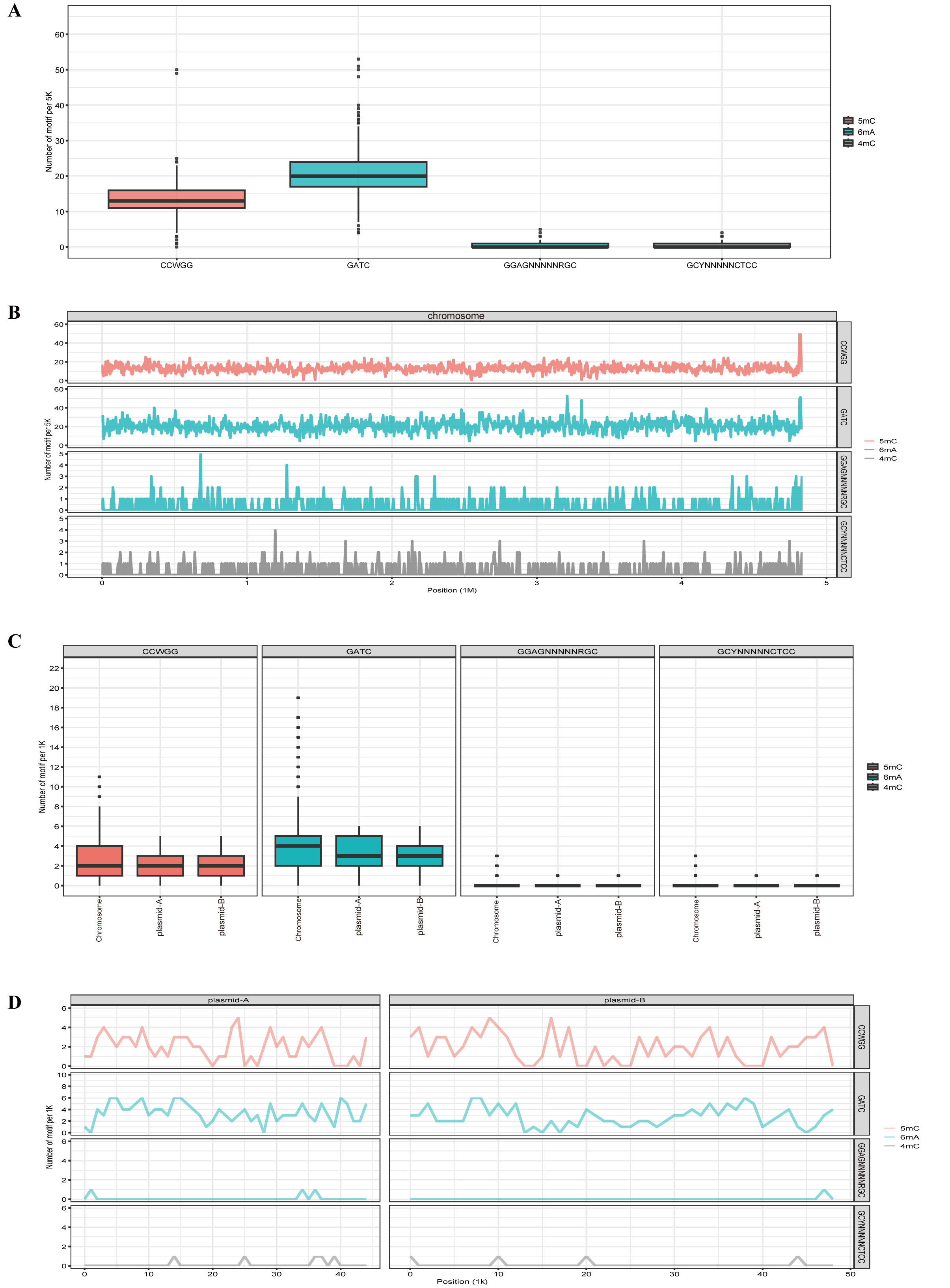

Supplement: Supplementary file 4 [file Image_3.TIF]
